# Neighborhood-level factors associated with COVID-19 vaccination rates: a case study in Chicago

**DOI:** 10.1186/s12889-024-18352-w

**Published:** 2024-03-25

**Authors:** Grace Keegan, Mengqi Zhu, Maria Paz, Hyojung Kang, Ajanta Patel, Arshiya A. Baig

**Affiliations:** 1 Pritzker School of Medicine, University of Chicago, 5841 S. Maryland Ave. MC 2007, 60637 Chicago, IL USA; 2 Department of Medicine, Section of General Internal Medicine, University of Chicago, Chicago, USA; 3 Department of Department of Kinesiology and Community Health, University of Illinois at Urbana-Champaign, Champaign, IL USA; 4 Chicago Department of Public Health, Chicago, IL USA

**Keywords:** COVID-19, Vaccination, Vaccine hesitancy, Health disparities, Primary care

## Abstract

**Introduction:**

Chicago’s deeply-rooted racial and socioeconomic residential segregation is a pattern mirrored in other major cities, making it a prototype for studying the uptake of public health interventions across the US. Residential segregation is related to availability of primary care, sense of community, and trust in the healthcare system, components which are essential in the response to crises like Covid-19 in which vaccine rollout was primarily community-based. We aimed to evaluate the association between rates of access to primary care and community-belonging with Covid-19 vaccination within Chicago’s neighborhoods.

**Methods:**

Data from Chicago Department of Public Health (12/2020-6/2022) on Covid-19 vaccination rates, race/ethnicity (% Black and % Hispanic/Latinx residents), age (% >65), gender (% female), socioeconomic status (% below the federal poverty line), access to needed care rate, and rate of self-reported sense of community-belonging on the neighborhood level were analyzed. Linear mixed models (LMMs) were used to study the impact of variables on vaccination; each neighborhood was added as a random effect to account for with-community association.

**Results:**

The average Covid-19 vaccination rates across Chicago’s neighborhoods was 79%, ranging from 37 to 100%, with median 81%. We found that Covid-19 vaccination rates were positively correlated with access to needed care (*p* < 0.001) and community-belonging (*p* < 0.001). Community areas that had lower vaccination rates had greater percentage of Black residents (*p* < 0.0001) and greater poverty rates (*p* < 0.0001). After adjusting for poverty, race, gender and age in the models, the association between vaccination rates and access to care or community-belonging were no longer significant, but % Black residents and poverty remained significant.

**Conclusions:**

Though access to needed primary care and community-belonging are correlated with vaccination rates, this association was not significant when controlling for demographic factors. The association between poverty, race and vaccination status remained significant, indicating that socioeconomic and racial disparities across Chicago drive Covid-19 vaccine recommendation adherence regardless of care access. Understanding how poverty, and its intersectional relation to race and primary care access, affects vaccination should be a priority for public health efforts broadly.

**Supplementary Information:**

The online version contains supplementary material available at 10.1186/s12889-024-18352-w.

## Introduction

Chicago is home to diverse residents living in 77 community areas (CAs) [1]. Chicago is also a city with deeply-rooted racial and socioeconomic residential segregation within these CAs, a pattern mirrored in other major cities, which makes it a prototype for studying public health interventions that are executed on a community level, most recently the COVID-19 vaccination rollout. As residential segregation is largely rooted in structural racism, structural racism is also evident in how the Covid-19 vaccine rollout began in Chicago and around the US, as more affluent and White neighborhoods received earlier access to and better supply of the Covid-19 vaccine [2, 3]. The injustice inherent in this process likely contributes to the disparities evident in COVID-19 vaccination rates, where the difference in vaccination rates between non-Hispanic Black and White residents is much higher in Chicago (13% higher in White residents) than the US overall (5% higher in White residents) [4, 5].

Primary care providers are traditionally important facilitators of public health interventions. A US survey data shows that 60% of people trust their primary care physician more than any other individual to provide information about the vaccine, implicating the important role of primary care in promoting vaccine adherence [6]. Previous studies have highlighted that metropolitan neighborhoods experiencing more segregation have less access to primary care physicians, implicating community segregation as a key player in vaccination rates [7]. It is also known that perceived community-belonging contributes to health-seeking behavior and preventative health care, including vaccinations [8]. There is a clear relationship between racial and socioeconomic segregation and access to care; however, no study has looked at access to needed care and perceived community belonging with adherence to Covid-19 vaccination on a community level, given the community-driven nature of the Covid-19 vaccine campaign and the inhibitive role that residential segregation may play in public health interventions.

In this study, we aimed to evaluate the association between access to care and perceived community belonging with community-level rates of COVID-19 vaccination, with respect to neighborhood-level variation in poverty rates and race, conceptualized as indicators of race-based residential segregation. Given the neighborhood-level pattern of the Covid-19 vaccine rollout in Chicago and in cities around the country, we used Chicago as a prototype to study these associations on a community-area level.

## Methods

Data were accessed on the Chicago Health Atlas and by requesting publicly accessible CA-level information on Covid-19 vaccination collected by the Chicago Department of Public Health (CDPH) from December 2020-June 2022. CAs were defined by the CDPH and are effectively interchangeable with the term “neighborhood”. These geographic units align similarly with zip codes and census tracts but are not the same, and more closely reflect community-based groupings rather than government designated areas. Chicago Health Atlas data were derived from the Healthy Chicago survey and census data; methodologies are available online [9, 10]. 

Our dependent outcome variable was vaccination completion rate, defined as the percent of responding households per CA reporting at least two doses of a two-shot series. Our independent predictor variables were the community adult rates reported of “received needed care” (percentage of households reporting they were able to obtain all “needed” health care) and “community-belonging” (percentage of respondents reporting having a sense of belonging to their community). These variables were self reported in the Healthy Chicago survey by a primary, adult respondent in the household. These independent variables are labeled as predictors given the original research question. Covariates, other demographic and independent variables of interest but not the main focus of the original research question, included community rates of race/ethnicity, age, gender, and poverty (% below the federal poverty line (≤ FPL)), uninsured rate.

Linear mixed models (LMMs) were used to assess the association between the predictors and vaccination completion rates. Each neighborhood was added as a random effect to account for with-community association. A univariate analysis was conducted before multivariate analysis controlling for demographic factors.

## Results

Table 1 summarizes the descriptive statistics for demographic variables of interest, including race, ethnicity, and poverty rates, and predictor variables, including access to needed care, primary care rate, and community belonging rate. The mean poverty rate across Chicago was 19.6% (Range = 3.51-53.9%), the average percent Non-Hispanic (NH) Black was 38.1% (Range = 0.37-96.5%), and the average percent Latinx was 19.6% (Range:3.51%=53.9%).

**Table 1:** Descriptive statistics of demographic, predictor, and outcome variables (*N* = 77 Community Areas)

**Demographics**	Mean	Range
***Race*** (%)		
*Non-Hispanic-Black*	38.1	0.37–96.5
*Non-Hispanic White*	28.0	0.82–80.8
**Ethnicity** (% Latinx)	26.0	0.85–89.2
**Poverty** (% <FPL)	19.6	3.51–53.9
**Age** (% Older than 65)	14.0	5.43–27.9
**Gender** (% Female)	52.2	42.0-64.8
**Access to Care and Community**		
Received Needed Care Rate (%)	82.0	56.4–95.0
Community Belonging Rate (%)	61.4	27.7–87.2
**Covid-19 Vaccination**		
Vaccine Series Completion Rate (%)	71.8	31.5–93.1

**Fig. 1:**
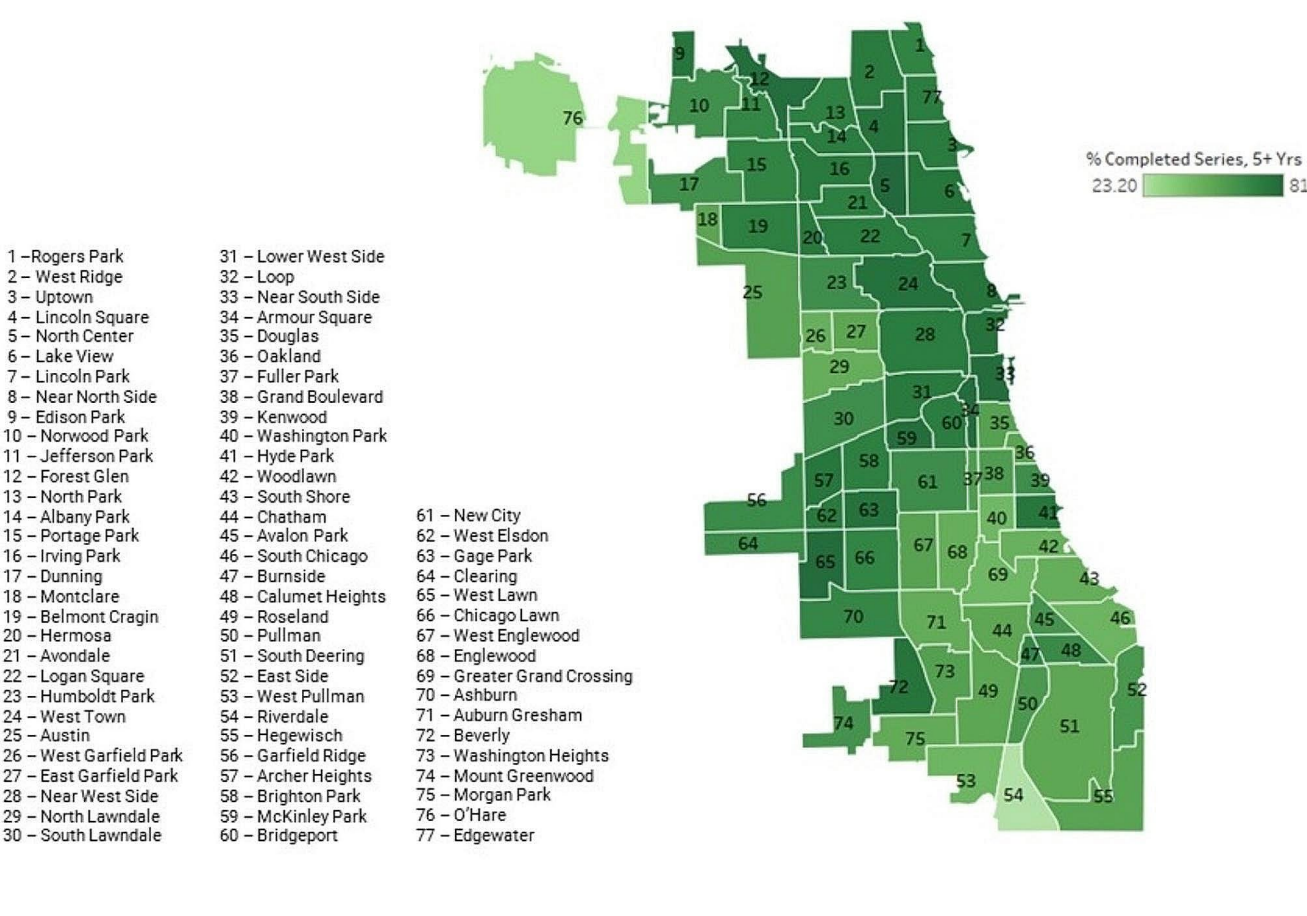
Heat map of chicago community areas and rate of COVID-19 vaccination

**Fig. 2:**
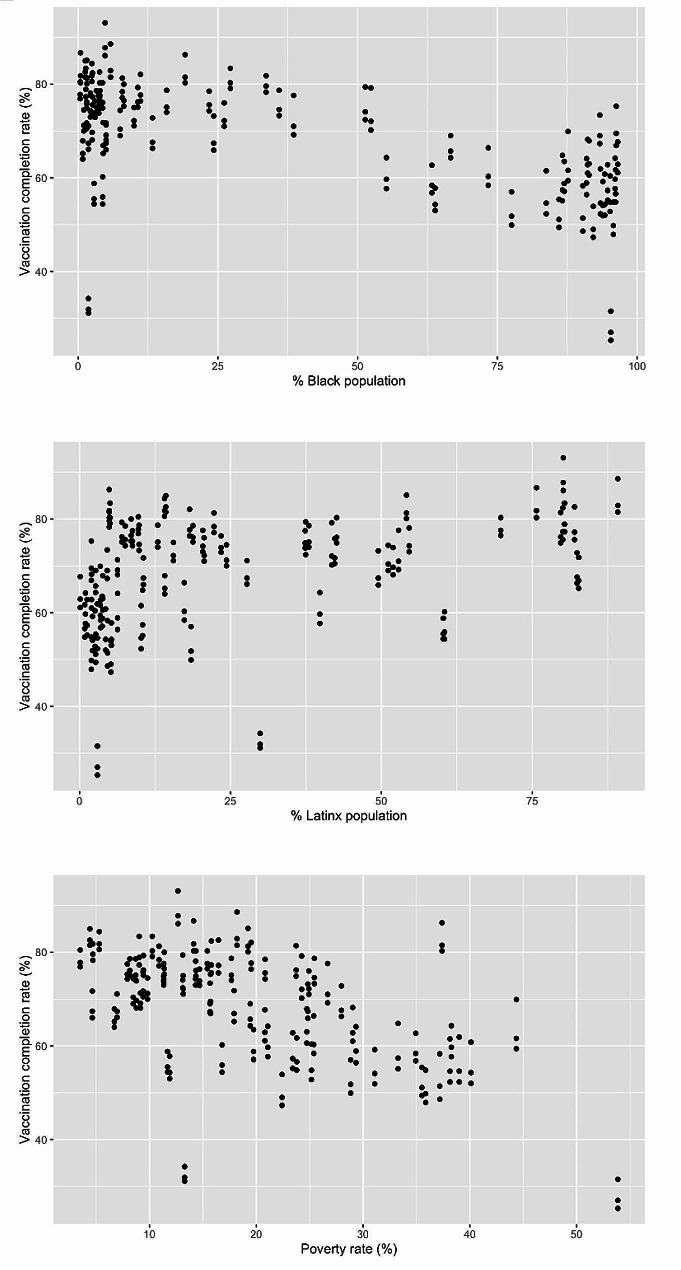
Correlation between race/ethnicity or poverty and vaccination rates

A heat map of Chicago’s community areas related to vaccination rates is included in Fig. 1. The average Covid-19 vaccination rate across Chicago’s neighborhoods was 71.8%, ranging from 31.5 to 93.1%. The association between vaccine completion and race, poverty, and ethnicity are demonstrated in Fig. 2. CAs that had lower vaccination rates had greater percentage of Black residents (*p* < 0.0001). Additionally, areas with a greater percentage of households living ≤ FPL were associated with lower vaccination rates (*p* < 0.0001). CAs with a greater percentage of people identifying as Latinx were associated with higher vaccination rates (*p* < 0.01). Univariate analysis results are summarized in Table 2.

**Table 2:** Univariate and multivariate analysis of care access, community belonging, and race/poverty with vaccination rates

	***Unadjusted***		***Adjusted***		***Adjusted***	
	Estimate	p-value	*Estimate*	*p-value*	*Estimate*	*p-value*
**Received Needed Care**	0.01	**< 0.001**	0.0044	0.08	---	**---**
**Community Belonging**	0.01	**< 0.001**	**---**	**---**	0.0005	0.82
**Poverty (%<FPL)**	-0.01	**< 0.0001**	-0.01	**< 0.0001**	-0.01	**< 0.01**
**Ethnicity (%Latinx)**	0.003	**< 0.01**	-0.003	**< 0.01**	-0.003	**< 0.01**
**Race (%NH-Black)**	-0.003	**< 0.0001**	0.003	**< 0.01**	0.004	**< 0.01**

On average, 77.6% of respondents received needed care (range 53.1 − 95%) and the average community-belonging rate was 43.1% (range 23.5 − 70.5%). We found in univariate analysis that Covid-19 vaccination rates were positively correlated with having access to needed care (*p* < 0.001) and sense of community-belonging (*p* < 0.001) (Table 2).

In multivariate models including residential composition of race, poverty, gender and age, the association between vaccination rates and access to needed care or community-belonging were no longer significant (*p* = 0.08 and 0.82, respectively). Residential racial composition (represented as %Black) (*p* < 0.01), ethnicity (represented as %Latinx) (*p* < 0.01) and poverty composition (*p* < 0.0001) remained significantly associated with community-level vaccination rates in the multivariable model for access to needed care. Additionally, residential racial composition (*p* < 0.01), ethnicity (*p* < 0.01), and poverty (*p* < 0.01) remained significantly associated with vaccination rates in the multivariable model for community belonging. In both of these multivariate analyses, the association between race (%Black residents) and poverty (%≤FPL) were negatively associated with vaccination rates, and ethnicity (%Latinx residents) was positively associated with vaccination rates. The multivariate results are also included in Table 2.

## **Discussion**

In this study, we found that areas with the greatest percentage of households living ≤ FPL and with greater percentage of NH Black residents had lower vaccination completion rates, and CAs with higher rates of Latinx residents had higher vaccination rates. Higher Covid-19 vaccination rates were associated with greater sense of community-belonging and better access to needed primary care. In multivariable models, the rates of community-belonging and access to needed care were not significant, but the percentage of NH Black residents and percentage living in poverty remained significantly negatively associated with Covid-19 vaccination rates, while percentage of Latinx people in a neighborhood remained significantly positively associated with vaccination. It should be noted that between April 2021 and July 2022, the national gap in vaccination rates between white and Black Americans decreased from 14 to 5%; we noted that that over a similar time-period, Chicago maintained an appreciable gap in vaccination rates by CA [4]. 

In our study, the socioeconomic and racial distribution by CA remained correlated with vaccination rate distribution after controlling for healthcare access variables. This indicates that access to needed care and perceived community belonging are associated with vaccination for COVID-19 on a neighborhood level only when considered alone, but poverty and racial composition of a community may be stronger predictors of vaccination rates by neighborhood. Community based strategy of COVID-19 vaccination has led to vaccination completion rates which vary significantly by the demographic makeup of a neighborhood, likely tied to the structural inequities which cause residential segregation and contributed to unjust rollout in vaccines. This underscores the need for social restructuring and investment in marginalized community areas resulting from segregation based on race, and the importance of community-based strategies to promote public health interventions in targeted communtiies at risk for low uptake of recommendations in the face of future pandemics [11]. 

These findings add greater complexity to the literature around access to care and vaccination rates, as primary care availability per capita is known to be associated with Covid-19 vaccination rates on a nationwide scale [12, 13]. While access to care is not significantly associated with Covid-19 vaccination rates by neighborhood in Chicago, trust may be explored in future studies as a possible mediator of this relationship. Historical marginalization of minority communities by the healthcare system and experiences of discrimination contribute to the barrier of trust between communities and healthcare providers, impacting acceptance of public health recommendations [14, 15]. One randomized control trial found that culturally-tailored messages addressing trust sent from primary care physicians to Black and Latinx patients resulted in a direct increase in vaccine uptake [13]. While trust in the healthcare system is most often explored from the perspective of the individual and related to individual health seeking behavior as in the case of the study by Lieu et al., it is also a community-level factor that may drive the strong association between residential race and poverty in our study.

Previous studies have also shown that sense of community-belonging can be a powerful driver in promoting preventive health behavior change such as vaccination [16]. While we did find associations between community-belonging, access to care, and vaccination rates, our findings indicate the structural drivers associated with socioeconomic disparities and racial segregation across Chicago may drive Covid-19 vaccine recommendation adherence through their strong association with “perceived community belonging” [17]. 

Finally, the Chicago CAs with the lowest vaccination rates and lowest rates of received needed care also had the highest rates of death due to Covid-19 in the city, a further reflection of disparities in care leading to Covid disparities [9]. The community-based approach to Covid-19 vaccine rollout fell short of addressing structural barriers and inequities that divide communities by race and socioeconomic status and may perhaps be strengthened by leveraging community voices and assets to promote more equitable vaccine distribution and uptake [17, 18]. Engaging community partners in this effort may address some individual and community-based factors, such as trust and health literacy, that lead to disparate vaccination rates [19]. Our findings also highlight the need for equitable vaccine practices informed by the outcomes of the Covid-19 vaccination campaigns, and for the use of vaccine equity checklists and continuous re-evaluation of targeted vaccination campaigns in response to future pandemics [17]. 

Our study has some limitations as the relationship between access to care, community belonging, and vaccination involves a complex set of demographic and structural issues only partially captured by our dataset. This study is limited by the use of community-level and not individual-level data, which may obscure some of the patterns we describe. We also are limited in statistical power by the 77 datapoints included as community areas. However, the purpose of this study was to draw conclusions from broader community-based patterns given the aforementioned community-level focus on the Covid-19 vaccine rollout, so these limitations do not preclude the associations described. And finally, we acknowledge that there was wide variation in the social patterning of access to vaccines and rates of vaccination across the study period, so our analysis of patterns of vaccine uptake within smaller time periods is limited.

## Conclusions

It is often assumed that improving access to primary care among communities with low uptake of public health interventions may improve health outcomes. However, our study finds that the socioeconomic makeup of a neighborhood and race-based residential segregation are more significant determining factors behind community-level adherence to public health interventions in a city with evident racial/ethnic clustering by CA. Understanding how socioeconomic deprivation within communities and how its intersectional relation to racial segregation relate to access to health care and adherence to vaccination should be a priority for public health efforts to improve the response to future public health crises.

### Electronic supplementary material

Below is the link to the electronic supplementary material.

Supplementary Material 1

## Data Availability

The datasets generated and/or analysed during the current study are available in the Chicago Health Atlas repository, https://chicagohealthatlas.org/download.
