# Supplementary material for: Neighborhood-level factors associated with COVID-19 vaccination rates: a case study in Chicago
Source: BMC Public Health. 2024 Mar 25;24:889. doi: 10.1186/s12889-024-18352-w (PMC10962191; doi:10.1186/s12889-024-18352-w)
Supplement: Supplementary file 1 — Supplementary Material 1 [file 12889_2024_18352_MOESM1_ESM.docx]

Appendix A: Correlation Table

|  | % completed series, 5+ yrs | PCP Rate | Access to Needed Care Rate | Routine Checkup Rate | Community Belonging | % Non-Hispanic White | % Non-Hispanic Black | % Hispanic or Latino | % Asian or Pacific Islander | % Older than 65 | Female | Poverty Rate |
| --- | --- | --- | --- | --- | --- | --- | --- | --- | --- | --- | --- | --- |
| % completed series, 5+ yrs | 1 |  |  |  |  |  |  |  |  |  |  |  |
| PCP Rate | 0.2736 | 1 |  |  |  |  |  |  |  |  |  |  |
| Access to Needed Care Rate | 0.3828 | 0.1382 | 1 |  |  |  |  |  |  |  |  |  |
| Routine Checkup Rate | -0.3877 | 0.3487 | -0.343 | 1 |  |  |  |  |  |  |  |  |
| Community Belonging | 0.429 | 0.3084 | 0.4675 | -0.2626 | 1 |  |  |  |  |  |  |  |
| % Non-Hispanic White | 0.4974 | 0.2747 | 0.5658 | -0.3649 | 0.6269 | 1 |  |  |  |  |  |  |
| % Non-Hispanic Black | -0.6833 | -0.1572 | -0.4029 | 0.5089 | -0.4988 | -0.7109 | 1 |  |  |  |  |  |
| % Hispanic or Latino | 0.3237 | -0.11 | 0.0003 | -0.3151 | 0.0176 | -0.079 | -0.6028 | 1 |  |  |  |  |
| % Asian or Pacific Islander | 0.449 | 0.1812 | 0.0383 | -0.1474 | 0.1699 | 0.263 | -0.3795 | -0.0771 | 1 |  |  |  |
| % Older than 65 | -0.1575 | 0.3344 | -0.0744 | 0.2305 | 0.1485 | -0.1149 | 0.2972 | -0.3398 | 0.0825 | 1 |  |  |
| Female | -0.5712 | -0.1394 | -0.2176 | 0.4629 | -0.381 | -0.3939 | 0.7068 | -0.5807 | -0.1584 | 0.123 | 1 |  |
| Poverty Rate | -0.6437 | -0.3531 | -0.5596 | 0.3722 | -0.6883 | -0.7265 | 0.6963 | -0.2495 | -0.0735 | -0.0118 | 0.506 | 1 |
